# Relationship between the methylenetetrahydrofolate reductase (*MTHFR*) rs1801133 SNP and serum homocysteine levels of Zhuang hypertensive patients in the central region of Guangxi

**DOI:** 10.1186/s40885-023-00250-9

**Published:** 2023-10-01

**Authors:** Xi-Jiang Hu, Mei-Ru Su, Bao-Wei Cao, Fa-Bang Ou, Rui-Xing Yin, An-De Luo

**Affiliations:** 1 Department of Cardiology, Laibin People’s Hospital, Laibin, China; 2 Department of Cardiology, Institute of Cardiovascular Diseases, The First Affiliated Hospital of Guangxi Medical University, Nanning, China

**Keywords:** Homocysteine, Hyperhomocysteinemia, H-type hypertension, Methylenetetrahydrofolate reductase, Single nucleotide polymorphism

## Abstract

**Background:**

The relationship between the methylenetetrahydrofolate reductase (*MTHFR*) single nucleotide polymorphism (SNP) and serum homocysteine (Hcy) levels or H-type hypertension in different populations is inconsistent. This study aimed to explore the association between the *MTHFR* rs1801133 SNP and serum Hcy levels of Zhuang hypertensive patients in the central region of Guangxi.

**Methods:**

A total of 606 Zhuang inpatients with essential hypertension were recruited in our hospital from August 2016 to December 2018. The patients were divided into H-type hypertension (Hcy > 10 µmol/L, n = 528) and non–H-type hypertension (Hcy ≤ 10 µmol/L, n = 78) groups. At the same time, an age- and sex-matched group of 379 subjects with normal physical examination in our hospital were selected as the control group. Blood biochemical measurements and genotyping of the *MTHFR* rs1801133 SNP were performed.

**Results:**

The prevalence of H-type hypertension was 87.13%. The levels of serum Hcy in patients with hypertension were higher than those in control group (14.20 ± 5.78 μmol/L vs. 11.97 ± 5.39 μmol/L, P < 0.001), especially in patients with H-type hypertension (15.08 ± 5.65 μmol/L, P < 0.001). The frequencies of TT genotype (22.73%) and T allele (46.21%) in patients with H-type hypertension were significantly higher than those in control group (11.35% and 30.47%, respectively) and non–H-type hypertension group (10.26% and 28.85%, respectively; P < 0.001 for all). Multivariate linear regression analysis showed that serum Hcy levels were significantly correlated with creatinine, low-density lipoprotein cholesterol, endogenous creatinine clearance rate, and the *MTHFR* rs1801133 genotypes in control group, while serum Hcy levels were significantly correlated with creatinine, triglyceride, low-density lipoprotein cholesterol, endogenous creatinine clearance rate, glycosylated hemoglobin, and the *MTHFR* rs1801133 genotypes in H-type hypertension group (P < 0.05–0.001). Serum Hcy levels in the T allele carriers were higher than those in the T allele noncarriers in both H-type hypertension and control groups.

**Conclusions:**

There was closely related between the *MTHFR* rs1801133 SNP and serum Hcy levels in Zhuang patients with H-type hypertension in the central region of Guangxi. The *MTHFR* SNP may be an important reason for the increase of serum Hcy levels in Zhuang patients with H-type hypertension in this region.

## Background

Essential hypertension (referred to as hypertension) is a common chronic cardiovascular disease that affects about a quarter of adults worldwide [1–3]. Long-term hypertension can not only cause hypertensive heart disease, but also lead to serious complications in several organs such as the heart, brain, kidneys, and fundus of the eye [4–6]. It is also the main culprit causing disability or death among middle-aged and elderly people [7–10]. With the rapid development of China’s economy, changes in lifestyle, intensification of population aging, and advancement of urbanization, hypertension has become a major public health problem [11–13]. The China Hypertension Survey (CHS) found that the crude prevalence rate of hypertension among residents aged ≥ 18 years in China from 2012 to 2015 was 27.9%, with an estimated number of 245 million people affected. The crude examination rate of prehypertension was 39.1%, and the estimated number of people with prehypertension was 435 million [14]. However, the prevalence of hypertension is not consistent across the country and among ethnic groups, generally higher in the north than in the south, and higher in cities than in rural areas [3, 13, 15]. Although the exact cause and pathogenesis of hypertension are not yet fully understood, it is widely believed that hypertension is a disease influenced by various factors such as lifestyle, diet, physical inactivity, genetic factors, and their interactions [16–21].

In the 1970s, it was noted that there was a significant relationship between elevated plasma homocysteine (Hcy) levels and the concomitant presence of hyperhomocysteinemia (HHcy; Hcy > 10 µmol/L) and various vascular diseases, such as atherosclerosis, hypertension, vascular calcification, aneurysm, and retinal vascular abnormalities. A previous Chinese population survey showed that the risk of cardiovascular and cerebrovascular events increased 2.3-fold in the population of Hcy > 9.47 µmol/L. The risk of death increased 2.4-fold in the population of Hcy > 11.84 µmol/L. Every 5 µmol/L increase in Hcy, the risk of stroke was also increased by 59% [22]. Therefore, some scholars refer to patients with both HHcy and primary hypertension as H-type hypertension [23]. About 80.0% of hypertensive patients in China were accompanied by HHcy [24, 25]. It is well-known that human plasma Hcy level is affected by gender and age [26, 27], races or ethnic groups [28–30], nutrition and dietary factors [31, 32], lifestyle [33], genetic factors [34–36], and diseases and drugs [37, 38]. The polymorphism or mutation of the key enzyme gene of Hcy metabolism such as cystathione-β-synthetase (*CBS*), methionine synthetase (*MS*), and 5,10-methylenetetrahydrofolate reductase (*MTHFR*) can change plasma Hcy levels. The human MTHFR gene is located at the end of the short arm of chromosome 1 (1p36.3). It is a coding gene for MTHFR. MTHFR is a key enzyme for folate and methionine metabolisms. *MTHFR* has a total length of 20.374 kb. There are 12 exons, and the length of messenger RNA is 7,150 base pair (bp), encoding a protein composed of 656 amino acid residues [39]. In the folate metabolism pathway, MTHFR can convert 5,10-methylenetetrahydrofolate into biologically functional 5-methyltetrahydrofolate; 5-methyltetrahydrofolate can further enter the methyl transmission pathway, indirectly provide methyl for DNA methylation and protein methylation through the process of Hcy remethylation and keep Hcy at a low-blood level. The *MTHFR* polymorphisms can lead to increased thermolability and impaired enzymatic activity. More than 15 species of polymorphisms and/or point mutation have been determined in the human *MTHFR*, and the most common of which is the *MTHFR* rs1801133 (C677T) SNP. The genotypes can divide into wild-type CC, heterozygous mutant CT, and homozygous mutant TT. Among them, the enzyme activity encoded by CC wild genotype is the strongest. When the wild type mutates into heterozygous CT or homozygous TT mutant, alanine in the gene expression enzyme structure is replaced by valine, which possesses a reduced overall enzyme activity to less than 30% of normal, resulting in serum Hcy level increased [40, 41], and Hcy cannot be converted into S-adenosylmethionine normally, thus affecting the metabolism of Hcy, and plasma Hcy level increases [30, 35, 36, 42–47]. However, this kind of relationship between the *MTHFR* SNP and serum Hcy levels is inconsistent in different populations [48].

There are 56 ethnic groups in China, and the Zhuang ethnic group is the most populous ethnic minority in China. According to the China Statistical Yearbook 2021, the population of the Zhuang ethnic group in China is 19,568,546. They mainly reside in the south of China. Guangxi Zhuang Autonomous Region (abbreviated as Guangxi) is the main distribution area of the Zhuang ethnic group, with a total of 14,207,100 people (87.81%) in 2000. The city Laibin is located in the central part of Guangxi and is also one of the main settlement cities for the Zhuang ethnic group. The clothing, dietary structure, customs, lifestyle, and genetic background of the Zhuang people are different from those of other ethnic groups. Therefore, the present study was undertaken to explore the relationship between the *MTHFR* rs1801133 SNP and serum Hcy levels or H-type hypertension in Zhuang ethnic group in the central region of Guangxi.

## Methods

### Ethics statement

The study was conducted in accordance with the Declaration of Helsinki. The study protocol was reviewed and approved by the Ethics Committee of Laibin People’s Hospital (No. 2,016,005), and all research subjects have signed informed consent forms.

### Subjects

All research subjects were hypertensive patients who were treated in our hospital from August 2016 to December 2018. The inclusion criteria were the following: (1) In accordance with the diagnostic criteria for hypertension in the “Chinese Guidelines for the Prevention and Treatment of Hypertension (revised 2018)”: in a quiet state without using antihypertensive drugs, the blood pressure values were continuously monitored three times on different days, with mean systolic blood pressure ≥ 140 mmHg (1 mmHg = 0.133 kPa) and/or diastolic blood pressure ≥ 90 mmHg. Having a history of hypertension and treated with antihypertensive drugs, although the blood pressure did not reach the above level during the physical examination, it was also diagnosed as hypertension [11–13, 16–21]. (2) All subjects are residents of the Zhuang ethnic group with three generations of ancestors in the central region of Guangxi Zhuang Autonomous Region, and their clinical data were complete. (3) Unrelated men or women aged ≥ 18 years. (4) With no severe chronic disease or systemic disease. (5) Willing to participate in the study. The exclusion criteria were as follows: (1) Secondary hypertension, white coat hypertension, and hypertensive crisis. (2) Malignant tumors, pregnant, or lactating women. (3) Thyroid disease, severe rheumatic immune system disease, and infectious disease. (4) Moderate to severe anemia, severe hematological diseases, and post–bone marrow transplantation. (5) Severe heart, liver, and kidney dysfunction. (6) Individuals with cognitive impairment who were unable to complete the questionnaire or had incomplete clinical data. A total of 606 hypertensive patients were included in this study. Among them, there were 186 male (30.69%) and 420 female patients (69.31%), aged 30 to 89 years, with an average age of 55.75 ± 9.94 years. The patients were divided into H-type hypertension group (H-type group, Hcy > 10 µmol/L) and non–H-type hypertension group (non–H-type group, Hcy ≤ 10 µmol/L) according to the serum Hcy levels. Another 379 normal persons who underwent normal physical examinations in our hospital during the same period were selected as the control group. Among them, there were 114 male (30.08%) and 265 female patients (69.92%), aged 29 to 93 years, with an average age of 54.96 ± 8.84 years. They were age- and sex-matched to the hypertensive group. The demographic characteristics and clinical data such as sex, age, smoking, drinking, medical history, and family history of the subjects were collected using predesigned tables, and the whole body physical examination was carried out, including measurement of height, weight, waist circumference, hip circumference, heart rate, and blood pressure.

### Estimation of sample size

The Quanto software ver. 1.2er. 1.2 (University of Southern California) was used to estimate the sample size [49, 50], and we proceed according to the following steps: (1) the prevalence of hypertension in the Chinese populations is nearly 27.9% (Kp); (2) selection of “case-control (matched)” and “gene only” from hypothesis; (3) the minor allele frequency of the rs1801133 SNP is 0.29, qA=√0.29 = 0.54; (4) specify the following values in the “outcome model” dialog: R_G_=1.8, K_p_= 27.9%; (5) choose 80% power, 0.05 significant level, and 2-sided alternative; and (6) the results indicated that 302, 98, and 214 case-control pairs for dominant, log-additive, and recessive models, respectively, were required for the desired power in the above setting. Therefore, the sample size in our study (case group, 606; control group, 379) was sufficient power for the statistical analyses.

### Blood biochemical measurements

A venous blood sample of 5 ml was collected after an overnight fasting in all subjects. A part of 3 ml was placed in a serum tube. After the blood coagulation, it was centrifuged at 3,500 r/min for 10 min, and then the serum was separated for biochemical testing. Another part of 2 ml was injected into an acid citrate dextrose (ACD) anticoagulant tube (containing 4.80 g/L citric acid, 14.70 g/L glucose, and 13.20 g/L trisodium citrate) and fully mixed for DNA extraction. Fasting blood glucose, serum creatinine, uric acid (UA), serum total cholesterol (TC), triglyceride (TG), high-density lipoprotein cholesterol (HDL-C), and low-density lipoprotein cholesterol (LDL-C) were detected by Hitachi 020 automatic biochemical analyzer (Hitachi). Serum Hcy levels were determined by using an enzymatic cycling method, and the detection kit includes reagents, calibrators, and quality control products were provided by Shanghai Kehua Biotechnology Co, Ltd (No. 20,030,916). The measurement was carried out on the Hitachi 020 automatic biochemical analyzer, and the experimenter strictly follows the instructions for operation. Briefly, the basic parameters of the biochemical analyzer were set as follows: the reaction method was the rate method; the main wavelength was 340 nm, and the secondary wavelength was 700 nm. The optical diameter of the colorimetric cup was 1.0 cm; the reaction direction was a descending reaction. Sample volume was 6.5 µl (blank tube with purified water, calibration tube with calibration substance). Reagent 1 was 120 µl, mixed well and incubated at 37 °C for 3 to 5 min, and then added reagent 2 of 33 µl, mixed well and incubated at 37 °C for 2.5 min and continuously monitor for approximately 2.5 min.

### Genotyping of the *MTHFR* rs1801133 SNP

The genomic DNA of the specimen was extracted from peripheral blood leukocytes using the phenol chloroform method, and the extracted DNA was stored at − 80°C until analysis. Genotyping of the *MTHFR* rs1801133 (C677T) SNP was performed on the Snapshot of next generation sequencing technology platform in Sangon Biotech Co, Ltd [17]. A HiSeqXTen sequencer (Illumina) was employed for SNP genotyping. The sense and antisense primers were 5’-CAAAGGCCACCCCGAAGC-3’ and 5’-AGGACGGTGCGGTGAGAGTG-3’, respectively.

### Statistical analysis

A database was established using EpiData ver. 3.02 (EpiData Association) and the data were analyzed using SPSS ver. 18.0 (SPSS Inc). The quantitative variables were represented by mean ± standard deviation, and the comparison between two groups was conducted using independent sample t-test. The enumeration data are expressed as percentage, and the comparison of different group rates was conducted using chi-square test. In order to evaluate the influencing factors of serum Hcy levels, multivariate linear regression analysis was also conducted on the H-type hypertension group and the control group, respectively. The independent variables include sex (female = 0, male = 1), age (years), height (cm), weight (kg), waist circumference (cm), hip circumference (cm), heart rate (beats/min), systolic blood pressure (mmHg), diastolic blood pressure (mmHg), urea nitrogen (mmol/L), creatinine (µmol/L), UA (µmol/L), endogenous creatinine clearance rate (ml/min), blood glucose (mmol/L), glycosylated hemoglobin (%), TC (mmol/L), TGs (mmol/L), HDL-C (mmol/L), LDL-C (mmol/L) and the *MTHFR* rs1801133 genotype (CC = 0, CT = 1, TT = 2). P < 0.05 indicates a statistically significant difference.

## Results

### General data between the hypertension and control groups

The comparison of general data and related biochemical parameters between the hypertension (including non–H-type and H-type hypertension) and the control groups is shown in Table 1. Among 606 hypertensive patients, H-type hypertension (Hcy > 10 µmol/L) was 528 patients (87.13%). The body weight, waist circumference, hip circumference, heart rate, systolic blood pressure, diastolic blood pressure, Hcy, UA, blood sugar, TG, and LDL-C levels were significantly higher in the hypertension group than those in the control group, whereas HDL-C levels were significantly lower in the hypertension group than those in the control group (P < 0.05–0.001). There was no significant difference in sex ratio, height, urea nitrogen, creatinine, endogenous creatinine clearance rate (Ccr), glycosylated hemoglobin, and TC levels between the two groups (all P > 0.05).

**Table 1:** Comparison of general data between the control and total hypertension, H-type hypertension, and non–H-type hypertension groups

Variable	Control (n = 379)	Hypertension			P-value		
		All (n = 606)	H-type (n = 528)	Non–H-type (n = 78)	P_1_	P_2_	P_3_
Sex					0.833	0.662	0.904
Male	114	186	162	24			
Female	265	420	366	54			
Age (yr)	55.96 ± 8.84	55.75 ± 9.94	56.06 ± 9.56	55.69 ± 12.08	0.433	0.094	0.204
Height (cm)	160.05 ± 6.53	160.12 ± 6.86	159.96 ± 6.70	161.19 ± 7.79	0.653	0.201	0.173
Weight (kg)	59.34 ± 8.92	61.32 ± 9.79	60.87 ± 8.67	64.39 ± 15.07	< 0.001	0.016	< 0.001
Waist circumference (cm)	81.81 ± 12.12	83.92 ± 10.64	83.94 ± 10.48	83.77 ± 11.76	0.004	0.908	0.193
Hip circumference (cm)	90.53 ± 13.07	92.28 ± 13.03	91.92 ± 12.50	94.69 ± 16.07	0.018	0.136	0.015
Heart rate (beats/min)	74.31 ± 10.14	77.03 ± 11.26	76.69 ± 11.43	79.31 ± 9.80	< 0.001	0.089	< 0.001
Systolic blood pressure (mmHg)	123.94 ± 10.33	158.23 ± 16.02	157.84 ± 15.01	160.89 ± 21.62	< 0.001	0.188	< 0.001
Diastolic blood pressure (mmHg)	72.94 ± 8.67	91.18 ± 12.81	90.71 ± 12.78	94.38 ± 12.62	< 0.001	0.038	< 0.001
Homocysteine (µmol/L)	11.97 ± 5.39	14.20 ± 5.78	15.08 ± 5.65	8.26 ± 1.65	< 0.001	< 0.001	< 0.001
Urea nitrogen (mmol/L)	5.54 ± 1.62	4.73 ± 1.15	4.85 ± 1.03	4.07 ± 1.20	0.289	< 0.001	< 0.001
Creatinine (µmol/L)	83.33 ± 18.46	82.40 ± 21.77	85.27 ± 15.35	64.39 ± 20.90	0.478	0.001	0.001
Uric acid (µmol/L)	381.55 ± 81.69	390.01 ± 58.98	381.36 ± 59.96	329.35 ± 78.20	0.035	0.016	0.366
Endogenous creatinine clearance rate (mL/min)	99.90 ± 14.47	99.36 ± 16.31	94.87 ± 11.44	115.85 ± 18.29	0.245	< 0.001	< 0.001
Blood glucose (mmol/L)	4.78 ± 0.64	4.99 ± 0.85	4.95 ± 0.67	5.41 ± 2.17	0.017	0.528	0.110
Glycosylated hemoglobin (%)	5.54 ± 0.46	5.56 ± 0.71	5.41 ± 0.53	5.96 ± 1.22	0.774	0.162	0.864
Total cholesterol (mmol/L)	5.27 ± 0.82	5.43 ± 0.81	5.51 ± 0.65	5.16 ± 1.03	0.204	0.006	0.084
Triglyceride (mmol/L)	1.56 ± 0.98	1.84 ± 1.15	2.03 ± 1.26	1.64 ± 1.04	0.001	0.203	0.536
High-density lipoprotein cholesterol (mmol/L)	1.56 ± 0.30	1.48 ± 0.37	1.52 ± 0.30	1.53 ± 0.45	< 0.001	0.267	0.003
Low-density lipoprotein cholesterol. (mmol/L)	3.45 ± 0.78	3.56 ± 0.97	3.79 ± 0.61	3.20 ± 1.04	0.024	< 0.001	0.015
Antihypertensive drugs	0	225 (37.13)	188 (35.61)	37 (47.44)^a)^	< 0.001	< 0.001	< 0.001

Data are presented as number only, mean ± standard deviation, or number (%). P_1_ values are compared between the control and total hypertension groups, P_2_ values are compared between the control and H-type hypertension groups, and P_3_ values are compared between the control and non–H-type hypertension groups

^a)^χ^2^ = 4.047, P = 0.043 in comparison with H-type hypertensive group

### General data between the control, H-type, and non–H-type groups

As also shown in Table 1, the body weight, diastolic blood pressure, Hcy, creatinine, TC, and LDL-C levels in the H-type hypertension group were significantly higher than those in the control group, while the levels of urea nitrogen, UA, and Ccr were significantly lower than those in the control group (P < 0.05–0.001). The body weight, hip circumference, heart rate, systolic blood pressure, diastolic blood pressure, and Ccr were significantly higher in the non–H-type hypertension group than in the control group, whereas the levels of Hcy, urea nitrogen, creatinine, HDL-C, and LDL-C were significantly lower in the non–H-type hypertension group than in the control group (P < 0.05–0.001).

### Genotype and allele frequencies among control, non–H-type, and H-type groups

**Fig. 1:**
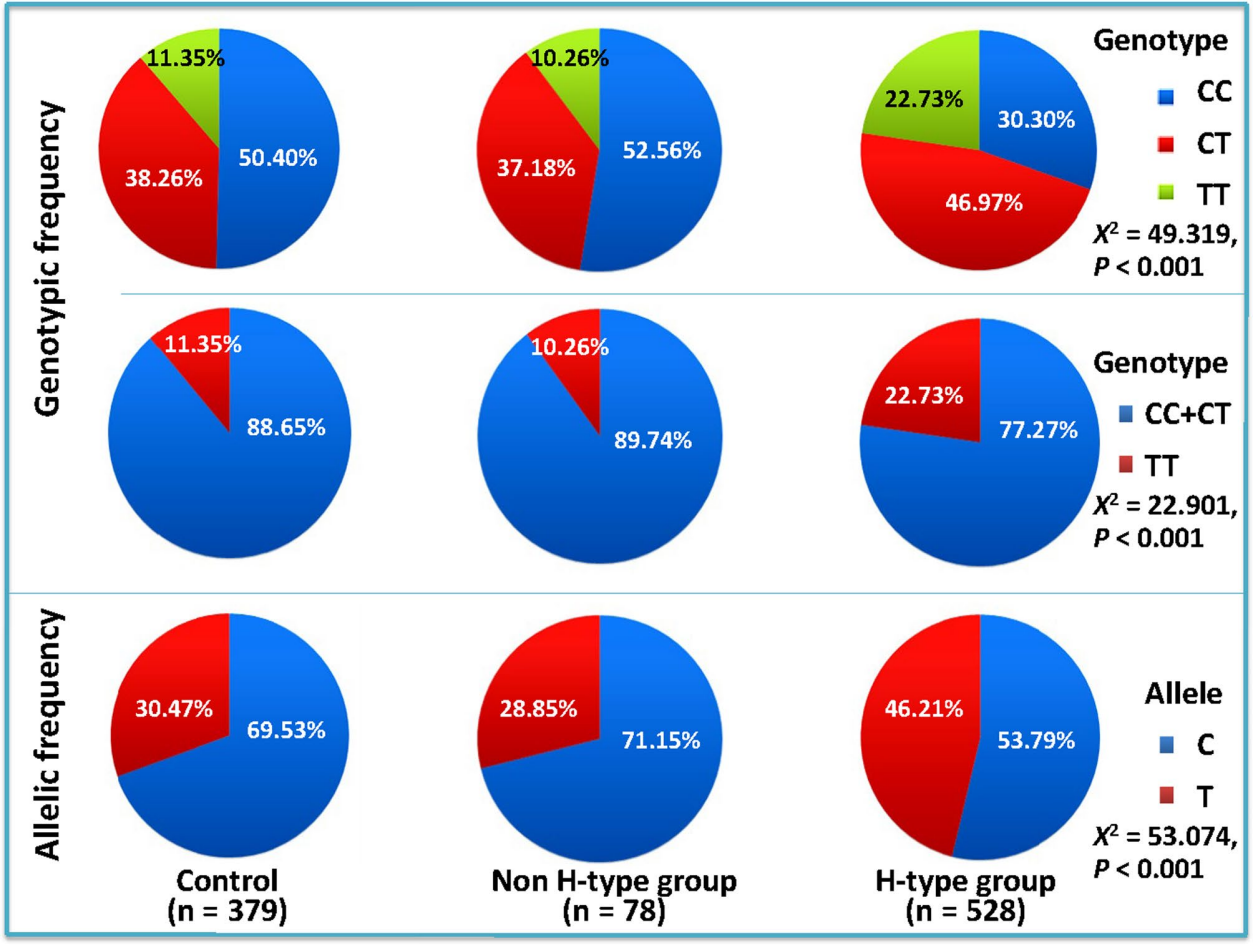
Comparison of genotypic and allelic frequencies of the methylenetetrahydrofolate reductase (*MTHFR*) rs1801133 single nucleotide polymorphism (SNP) in the control, non–H-type hypertension, and H-type hypertension groups. The differences in the genotypic and allelic frequencies between controls and hypertensive patients were determined by the chi-square test

The comparison of genotype and allele frequencies among the control, non–H-type and H-type hypertension groups is shown in Fig. 1. The genotype distribution of the three groups was consistent with the Hardy-Weinberg equilibrium (all P > 0.05). There were statistically significant differences in genotype and allele frequencies among the three groups (all P < 0.001). The frequencies of TT genotype (22.73%) and T allele (46.21%) in the H-type hypertension group were significantly higher than those in the control group (11.35% and 30.47%, respectively) and the non–H-type hypertension group (10.26% and 28.85%, respectively).

### Influencing factors of serum hcy levels in the control and H-type groups

The influencing factors of serum Hcy levels in the control group and H-type hypertension group are shown in Table 2. The results of multivariate linear regression analysis showed that the influencing factors of serum Hcy levels in the control group were creatinine, LDL-C, Ccr, and the *MTHFR* rs1801133 (C677T) genotypes. The influencing factors of serum Hcy levels in the H-type hypertension group include creatinine, TGs, LDL-C, Ccr, glycosylated hemoglobin, and the *MTHFR* rs1801133 (C677T) genotypes. In both groups of study subjects, serum Hcy levels of T allele carriers (TT and CT genotypes) were higher than those of T allele noncarriers (CC genotypes).

**Table 2:** Influencing factors of serum Hcy levels in the control and H-type hypertension groups

Variable	Standardized coefficient	P-value	95% Confidence interval
Control group			
Creatinine (µmol/L)	0.165	0.032	0.004 to 0.085
LDL-C (mmol/L)	0.286	0.021	0.280 to 3.412
Ccr (ml/min)	–0.273	< 0.001	–0.116 to − 0.036
*MTHFR* rs1801133 genotype	0.258	0.013	0.095 to 0.190
H-type group			
Creatinine (µmol/L)	0.184	0.002	0.019 to 0.083
Triglyceride(mmol/L)	0.167	0.001	0.272 to 1.036
LDL-C (mmol/L)	0.249	0.005	0.501 to 2.874
Ccr (ml/min)	–0.190	< 0.001	–0.092 to − 0.029
Glycosylated hemoglobin (%)	–0.234	< 0.001	–2.593 to − 0.890
*MTHFR* rs1801133 genotype	0.226	0.001	0.045 to 0.143

LDL-C, low-density lipoprotein cholesterol; Ccr, endogenous creatinine clearance rate; THFR, methylenetetrahydrofolate reductase

## Discussion

In the present study, we detected the relationship between the *MTHFR* rs1801133 SNP and serum Hcy levels in Zhuang hypertensive patients from Laibin city of Guangxi. The results showed that the prevalence of H-type hypertension in Zhuang ethnic group was 87.13% in Laibin city, which is slightly higher than in the Shanghai region of China, the prevalence of H-type hypertension among elderly patients in Shanghai region was 80.0% [25]. However, the previously reported prevalence of H-type hypertension in China was around 75% [23]. These differences may be related to regional and demographic differences. It has been found that plasma Hcy levels in Chinese people were higher than those in the West. The western regions of China were higher than those of the eastern coastal areas, the northern regions were higher than those of the southern regions, and the urban areas were higher than those of the rural areas. This difference in serum Hcy levels was similar to the epidemiological distribution of primary hypertension [28]. In addition, there were also sex- and age-related differences in plasma Hcy levels. The Hcy level was significantly higher in men than in women in each age range from 20 to over 80 years, and the trend did not abate with age. The Hcy level first decreased and then increased, being lowest at 30 to 50 years of age and significantly increased after 50 years of age [26, 27, 51]. The prevalence of HHcy was higher in men than in women, and the incidence rate of H-type hypertension was also higher in people ≥ 55 years old than those < 55 years old [26, 27].

In the current study, we found that serum Hcy levels were significantly higher in hypertensive patients than in the control group (14.20 ± 5.78 µmol/L vs. 11.97 ± 5.39 µmol/L, P < 0.001), especially in the patients with H-type hypertension (15.08 ± 5.65 µmol/L, P < 0.001). However, serum Hcy levels in non–H-type hypertension patients (8.26 ± 1.65 µmol/L) were actually lower than those in the control group (P < 0.001). The reason for this discrepancy is not yet clear. We speculate that it may be related to the impact of therapeutic drugs. It has been reported that plasma Hcy levels were influenced by some drugs, such as antiepileptics, anticonvulsants, methotrexate, metformin, azalipin, carbamazepine, and diuretics. These medicines can cause an increase in plasma Hcy levels by interfering with folate and methionine metabolism, respectively. Conversely, some drugs such as oral contraceptives, hormone replacement therapy, antirheumatic drugs, penicillamine, acetylcysteine, and atorvastatin calcium tablets can reduce plasma Hcy levels [37, 38, 52, 53]. Unfortunately, some of our research subjects were taking some antihypertensive drugs such as diuretics, β-blockers, calcium antagonists, angiotensin-converting enzyme inhibitors, or angiotensin receptor blockers. But they did not provide detailed medication and dosage records, and we cannot determine the degree of impact of these drugs on serum Hcy levels. This is also our work deficiency. In addition, we also found that the frequencies of the *MTHFR* rs1801133 TT genotype and T allele were slightly lower in non–H-type hypertensive patients than in control group. Perhaps this may be the real reason why serum Hcy level was lower in non–H-type hypertensive patients than in the control group. In the present study, we revealed that the levels of body weight, hip circumference, heart rate, systolic blood pressure, diastolic blood pressure, and Ccr were significantly higher in the non–H-type hypertension than in the control groups. It seems that in non–H-type hypertensive individuals, elevated blood pressure may be related to obesity. It is widely recognized that overweight and obesity are closely associated with hypertension [54]. Weight gain was usually associated with a corresponding increase in blood pressure [55]. The prevalence of hypertension in children was estimated to be 3–14% for normal weight children and 11–30% for obese children [56]. Excess adiposity was the single most powerful risk factor for higher blood pressure and contributed to more than half of the risk for developing hypertension [57].

The human cannot directly ingest Hcy from food and cannot synthesize it in the body. It is an important intermediate product in the essential amino acid methionine cycle and cysteine metabolism. It can be metabolized through the following pathways: (1) remathylation, in which the key enzyme for the formation of 5-methyltetrahydrofolate is the MTHFR; (2) alternative pathways for methylation; (3) transvulcanization; (4) release to extracellular fluid; and (5) oxidation (self-oxidation under the catalysis of metal ions such as iron or calcium). From here we see that any abnormality in the Hcy metabolic pathway or the lack of various enzymes and cofactors involved in Hcy metabolism will result in abnormal Hcy metabolism, leading to its accumulation in the body and causing HHcy. Defects and variations in key enzyme genes involved in Hcy metabolism were the most common genetic factors affecting plasma Hcy concentration. The *MTHFR* polymorphisms can lead to a decrease in MTHFR activity and an increase in plasma Hcy levels [30, 35, 36, 42–47]. More than 15 types of *MTHFR* SNPs have been found, with the most common being the *MTHFR* rs1801133 (C677T) SNP. The wild-type CC genotype encodes the strongest MTHFR enzyme activity. When it mutates into a heterozygous CT or homozygous TT mutant, alanine is replaced by valine in the gene expression enzyme structure, resulting in a decrease in enzyme activity. Hcy cannot be converted into S-adenosylmethionine normally, thereby affecting its metabolism and increasing plasma Hcy levels [30, 35, 36, 42–47]. In the current study, we also found that the frequencies of the *MTHFR* rs1801133 TT genotype (22.73%) and T allele (46.21%) in Zhuang patients with H-type hypertension in the central region of Guangxi were significantly higher than those in the control group (11.35% and 30.47%, respectively) and the non–H-type hypertension group (10.26% and 28.85%, respectively). The results of multivariate linear regression analysis showed that there was a significant correlation between serum Hcy levels and the *MTHFR* rs1801133 genotypes in both control and H-type hypertension groups. Serum Hcy levels in the T allele carriers (TT and CT genotypes) were higher than those in the T allele non-carriers (CC genotype). These findings suggest that the decrease in MTHFR activity caused by the *MTHFR* SNP may be an important reason for the increased plasma Hcy levels. It is worth noting that the efficacy of folic acid supplementation was related to the genotypes of *MTHFR* C677T. Qin et al. [58, 59] have demonstrated that the *MTHFR* C677T polymorphism can not only affect serum Hcy and folate levels at baseline and post–folic acid treatment, but also can modify therapeutic responses to various dosages of folic acid supplementation. These results may provide new clues and experimental basis for the precise treatment of hypertension patients based on their genotypes, and also provide new ways to achieve personalized accurate early warning, diagnosis and intervention of metabolic disorder such as hypertension in the future.

However, some authors found that there was no relationship between the *MTHFR* C677T polymorphism and the risk of H-type hypertension in Guangxi Zhuang ethnic group [48]. The reason for this diversity is not yet clear. We speculate that it may be related to the following factors: (1) In the previous study [48], the number of the study patients was too small, with only 185 hypertensive patients in all, of which only 76 persons were H-type hypertensive patients. The subjects with TT genotype were only six (7.89%) in H-type hypertension and five (4.59%) in non–H-type hypertension. (2) H-type hypertension in the study was defined as Hcy > 15 µmol/L, which significantly reduced the number of cases of H-type hypertension (n = 76) compared to the number of cases of non–H-type hypertension (n = 109). Therefore, further research is needed to confirm the relationship between the *MTHFR* C677T SNP and the risk of H-type hypertension in different races or ethnic groups.

There are several potential limitations in the present study. First, the sample size of non–H-type hypertension group is a bit small. Second, there was different in sex ratio of the study populations, the number of women was greater than that of men. Third, drug information did not describe in detail in this study, and the effects of drugs on serum Hcy levels and the *MTHFR* SNP are still needed to research. Finally, the interactions of gene-gene, gene-environment, and environment-environment on serum Hcy levels and the *MTHFR* SNP remain to be determined.

## Conclusions

In conclusion, this study shows that the prevalence of H-type hypertension in Laibin, a city in the central region of Guangxi, China is very high. There was also closely association between the *MTHFR* rs1801133 SNP and H-type hypertension in this ethnic group. The *MTHFR* SNP may be an important reason for the increased serum Hcy levels and the prevalence of H-type hypertension in this region. Therefore, early identification, monitoring, intervention, and management of H-type hypertension should be carried out in this region.

## Data Availability

The datasets used and/or analyzed during the current study are available from the corresponding author on reasonable request.

## Abbreviations

ACD: Acid citrate dextrose
bp: Base pair
CBS: Cystathione-β-synthetase
Ccr: Endogenous creatinine clearance rate
CHS: China Hypertension Survey
Hcy: Homocysteine
HDL-C: High-density lipoprotein cholesterol
HHcy: Hyperhomocysteinemia
LDL-C: Low-density lipoprotein cholesterol
MS: Methionine synthetase
MTHFR: Methylenetetrahydrofolate reductase
SNP: Single nucleotide polymorphism
TC: Total cholesterol
TG: Triglyceride
UA: Uric acid

## References

[1] Patel P, Ordunez P, DiPette D, Escobar MC, Hassell T, Wyss F (2016). Improved blood pressure control to reduce cardiovascular disease morbidity and mortality: the standardized hypertension treatment and prevention project. J Clin Hypertens (Greenwich).

[2] Wang X, Bots ML, Yang F, Hoes AW, Vaartjes I (2014). Prevalence of hypertension in China: a systematic review and meta-regression analysis of trends and regional differences. J Hypertens.

[3] Gao Y, Chen G, Tian H, Lin L, Lu J, Weng J (2013). Prevalence of hypertension in China: a cross-sectional study. PLoS ONE.

[4] Oliveras A, de la Sierra A (2014). Resistant hypertension: patient characteristics, risk factors, co-morbidities and outcomes. J Hum Hypertens.

[5] Piskorz D (2020). Hypertensive mediated organ damage and hypertension management: how to assess beneficial effects of antihypertensive treatments?. High Blood Press Cardiovasc Prev.

[6] Wenzel UO, Bode M, Köhl J, Ehmke H (2017). A pathogenic role of complement in arterial hypertension and hypertensive end organ damage. Am J Physiol Heart Circ Physiol.

[7] Ruixing Y, Jiaqiang D, Dezhai Y, Weixiong L, Shangling P, Jinzhen W (2006). Effects of demographic characteristics, health-related behaviors and lifestyle factors on the prevalence of hypertension for the middle-aged and elderly in the Guangxi Hei Yi Zhuang and Han populations. Kidney Blood Press Res.

[8] Yin R, Li H, Wu J, Lin W, Yang D, Pan S (2007). Effects of alcohol consumption and other lifestyle behaviors on blood pressure for the middle-aged and elderly in the Guangxi Hei Yi Zhuang and Han populations. Alcohol.

[9] Ruixing Y, Weixiong L, Hanjun Y, Dezhai Y, Shuquan L, Shangling P (2008). Diet, lifestyle, and blood pressure of the middle-aged and elderly in the Guangxi Bai Ku Yao and Han populations. Am J Hypertens.

[10] Wang YJ, Li ZX, Gu HQ, Zhai Y, Zhou Q, Jiang Y, China National Clinical Research Center for Neurological Diseases, the Chinese Stroke Association (2022). China Stroke Statistics: an update on the 2019 report from the National Center for Healthcare Quality Management in Neurological Diseases. Stroke Vasc Neurol.

[11] Bin Y, Meng EJ, Ya YX, Yin RX, Liu WY, Zhang L (2017). Prevalence, awareness, treatment, control and the risk factors of hypertension in the chinese Maonan and Han ethnic groups. Int J Clin Exp Med.

[12] Hu XJ, Yin RX, Li H, Shi YL, Wang YM, Wei MF (2016). Prevalence of hypertension and its risk factors in the chinese Mulao and Han populations. Int J Clin Exp Med.

[13] Xie RB, Liao PJ, Yin RX, Hu XJ, Huang J, Wei DX (2015). Prevalence of hypertension and associated risk factors in Chinese Jing compared with Mulao populations. J Int Med Res.

[14] Wang Z, Chen Z, Zhang L, Wang X, Hao G, Zhang Z (2018). Status of hypertension in China: results from the China Hypertension Survey, 2012–2015. Circulation.

[15] Reynolds K, Gu D, Muntner P, Wu X, Chen J, Huang G (2003). Geographic variations in the prevalence, awareness, treatment and control of hypertension in China. J Hypertens.

[16] Ruixing Y, Hui L, Jinzhen W, Weixiong L, Dezhai Y, Shangling P (2009). Association of diet and lifestyle with blood pressure in the Guangxi Hei Yi Zhuang and Han populations. Public Health Nutr.

[17] Wei BL, Yin RX, Liu CX, Deng GX, Guan YZ, Zheng PF (2021). CYP17A1-ATP2B1 SNPs and gene-gene and gene-environment interactions on essential hypertension. Front Cardiovasc Med.

[18] Yin RX, Aung LH, Long XJ, Yan TT, Cao XL, Huang F (2015). Interactions of several genetic polymorphisms and alcohol consumption on blood pressure levels. BioFactors.

[19] Yin RX, Wu DF, Aung LH, Yan TT, Cao XL, Long XJ (2012). Several lipid-related gene polymorphisms interact with overweight/obesity to modulate blood pressure levels. Int J Mol Sci.

[20] Yin RX, Wu DF, Wu JZ, Cao XL, Aung LH, Miao L (2012). Interactions of several lipid-related gene polymorphisms and cigarette smoking on blood pressure levels. Int J Biol Sci.

[21] Yin RX, Wu JZ, Liu WY, Wu DF, Cao XL, Miao L (2012). Association of several lipid-related gene polymorphisms and blood pressure variation in the Bai Ku Yao population. Am J Hypertens.

[22] Li J, Jiang S, Zhang Y, Tang G, Wang Y, Mao G (2015). H-type hypertension and risk of stroke in chinese adults: a prospective, nested case-control study. J Transl Int Med.

[23] Hu DY, Xu XP (2008). Prevention of stroke relies on valid control H type hypertension. Zhonghua nei ke za zhi.

[24] Huo Y, Li J, Qin X, Huang Y, Wang X, Gottesman RF (2015). Efficacy of folic acid therapy in primary prevention of stroke among adults with hypertension in China: the CSPPT randomized clinical trial. JAMA.

[25] Qian XL, Cao H, Zhang J, Gu ZH, Tang WQ, Shen L (2021). The prevalence, relative risk factors and MTHFR C677T genotype of H type hypertension of the elderly hypertensives in Shanghai, China: a cross-section study: prevalence of H type hypertension. BMC Cardiovasc Disord.

[26] Li T, Wang C, Ma L (2022). Analysis of clinical characteristics and influencing factors for H-type hypertension complicated with other chronic diseases in a community in Beijing. Evid Based Complement Alternat Med.

[27] Wang Y, Li X, Qin X, Cai Y, He M, Sun L (2013). Prevalence of hyperhomocysteinaemia and its major determinants in rural chinese hypertensive patients aged 45–75 years. Br J Nutr.

[28] Liang Z, Fan FF, Zhang Y, Qin XH, Li JP, Huo Y (2022). Rate and characteristics of H-type hypertension in chinese hypertensive population and comparison with american population. Beijing Da Xue Xue Bao Yi Xue Ban.

[29] du Plessis JP, Lammertyn L, Schutte AE, Nienaber-Rousseau C (2022). H-type hypertension among Black South Africans and the relationship between homocysteine, its genetic determinants and estimates of vascular function. J Cardiovasc Dev Dis.

[30] Liao S, Guo S, Ma R, He J, Yan Y, Zhang X (2022). Association between methylenetetrahydrofolate reductase (MTHFR) C677T polymorphism and H-type hypertension: a systematic review and meta-analysis. Ann Hum Genet.

[31] Stea TH, Mansoor MA, Wandel M, Uglem S, Frølich W (2008). Changes in predictors and status of homocysteine in young male adults after a dietary intervention with vegetables, fruits and bread. Eur J Nutr.

[32] Wang W, Ji P, Wang Y, Guo H, Bian R, Xu J (2018). Prevalence of hyperhomocysteinemia and its associated factors in patients with primary hypertension in chinese urban communities: a cross-sectional study from Nanjing. Clin Exp Hypertens.

[33] de Bree A, Verschuren WM, Blom HJ, Kromhout D (2001). Lifestyle factors and plasma homocysteine concentrations in a general population sample. Am J Epidemiol.

[34] Kripps KA, Sremba L, Larson AA, Van Hove JL, Nguyen H, Wright EL (2022). Methionine synthase deficiency: variable clinical presentation and benefit of early diagnosis and treatment. J Inherit Metab Dis.

[35] Zhang C, Li J, Zhou J, Zheng Q, Dong R, Xing E (2022). Effect of MTHFRC677 T gene polymorphism on early morning blood pressure in elderly female patients with H-type hypertension. Contrast Media Mol Imaging.

[36] Zhang C, Dou Z, Zhao C, Li J, Xin Q, Feng Y (2022). Analysis of the correlation between the distribution of MTHFR gene and the severity and renal function of elderly patients with H-type hypertension. J Healthc Eng.

[37] Dai C, Fei Y, Li J, Shi Y, Yang X (2021). A novel review of homocysteine and pregnancy complications. Biomed Res Int.

[38] Zhang S, Wang T, Wang H, Tang J, Hou A, Yan X (2022). Effects of individualized administration of folic acid on prothrombotic state and vascular endothelial function with H-type hypertension: a double-blinded, randomized clinical cohort study. Med (Baltim).

[39] Ou CY, Stevenson RE, Brown VK, Schwartz CE, Allen WP, Khoury MJ (1996). 5,10 methylenetetrahydrofolate reductase genetic polymorphism as a risk factor for neural tube defects. Am J Med Genet.

[40] Regland B, Blennow K, Germgård T, Koch-Schmidt AC, Gottfries CG (1999). The role of the polymorphic genes apolipoprotein E and methylene- tetrahydrofolate reductase in the development of dementia of the Alzheimer type. Dement Geriatr Cogn Disord.

[41] Nishiyama M, Kato Y, Hashimoto M, Yukawa S, Omori K, Apolipoprotein E (2000). Methylenetetrahydrofolate reductase (MTHFR) mutation and the risk of senile dementia: an epidemiological study using the polymerase chain reaction (PCR) method. J Epidemiol.

[42] Fu L, Li YN, Luo D, Deng S, Wu B, Hu YQ (2019). Evidence on the causal link between homocysteine and hypertension from a meta-analysis of 40 173 individuals implementing mendelian randomization. J Clin Hypertens (Greenwich).

[43] Song J, Hou J, Zhao Q, Liu X, Guo Q, Yin D (2020). Polymorphism of MTHFR C677T gene and the associations with the severity of essential hypertension in Northern Chinese population. Int J Hypertens.

[44] Ornosa-Martín G, Fernandez-Ballart JD, Ceruelo S, Ríos L, Ueland PM, Meyer K (2020). Homocysteine, the methylenetetrahydrofolate reductase 677 C > T polymorphism and hypertension: effect modifiers by lifestyle factors and population subgroups. Br J Nutr.

[45] Huang LW, Li LL, Li J, Chen XR, Yu M (2022). Association of the methylenetetrahydrofolate reductase (MTHFR) gene variant C677T with serum homocysteine levels and the severity of ischaemic stroke: a case-control study in the southwest of China. J Int Med Res.

[46] Yun L, Xu R, Li G, Yao Y, Li J, Cong D (2015). Homocysteine and the C677T gene polymorphism of its key metabolic enzyme MTHFR are risk factors of early renal damage in hypertension in a chinese Han population. Med (Baltim).

[47] Mabhida SE, Muhamed B, Sharma JR, Apalata T, Nomatshila S, Mabasa L (2022). Methylenetetrahydrofolate reductase polymorphism (rs1801133) and the risk of hypertension among african populations: a narrative synthesis of literature. Genes (Basel).

[48] Huang LQ, Wu CX, Wei HQ, Xu G (2020). Clinical characteristics of H-type hypertension and its relationship with the MTHFR C677T polymorphism in a Zhuang population from Guangxi, China. J Clin Lab Anal.

[49] Gauderman WJ (2002). Sample size requirements for association studies of gene-gene interaction. Am J Epidemiol.

[50] Burton PR, Hansell AL, Fortier I, Manolio TA, Khoury MJ, Little J (2009). Size matters: just how big is BIG?: quantifying realistic sample size requirements for human genome epidemiology. Int J Epidemiol.

[51] Xu R, Huang F, Wang Y, Liu Q, Lv Y, Zhang Q (2020). Gender- and age-related differences in homocysteine concentration: a cross-sectional study of the general population of China. Sci Rep.

[52] Li P, Li J, Zhao H, Wang M (2018). The study status of homocysteine in patients with hypertension disorder complicating pregnancy. Contemp Med.

[53] Na K, Yu P, Zhang G (2010). Attention should be paid to the relationship between homocysteinemia and hypertension. Chin J Clin.

[54] Gelber RP, Gaziano JM, Manson JE, Buring JE, Sesso HD (2007). A prospective study of body mass index and the risk of developing hypertension in men. Am J Hypertens.

[55] Davy KP, Hall JE (2004). Obesity and hypertension: two epidemics or one?. Am J Physiol Regul Integr Comp Physiol.

[56] Willig AL, Casazza K, Dulin-Keita A, Franklin FA, Amaya M, Fernandez JR (2010). Adjusting adiposity and body weight measurements for height alters the relationship with blood pressure in children. Am J Hypertens.

[57] Lurbe E, Cifkova R, Cruickshank JK, Dillon MJ, Ferreira I, Invitti C (2010). Management of high blood pressure in children and adolescents: recommendations of the European Society of hypertension. An Pediatr (Barc).

[58] Qin X, Li J, Cui Y, Liu Z, Zhao Z, Ge J (2012). MTHFR C677T and MTR A2756G polymorphisms and the homocysteine lowering efficacy of different doses of folic acid in hypertensive chinese adults. Nutr J.

[59] Qin X, Li J, Cui Y, Liu Z, Zhao Z, Ge J (2012). Effect of folic acid intervention on the change of serum folate level in hypertensive chinese adults: do methylenetetrahydrofolate reductase and methionine synthase gene polymorphisms affect therapeutic responses?. Pharmacogenet Genomics.

